# Somatic Symptoms Are Associated With Elevated Blood Pressure and Epstein–Barr Virus Antibodies Among Shuar of the Ecuadorian Amazon

**DOI:** 10.1002/ajhb.24191

**Published:** 2024-11-29

**Authors:** Paula S. Tallman, Rebecca A. Seligman, Felicia C. Madimenos, Melissa A. Liebert, Tara J. Cepon‐Robins, J. Josh Snodgrass, Thomas W. McDade, Lawrence S. Sugiyama

**Affiliations:** ^1^ Department of Anthropology Loyola University Chicago Chicago Illinois USA; ^2^ The Keller Science Action Center Field Museum of Natural History Chicago Illinois USA; ^3^ Social Welfare Graduate Program Universitas Padjadjaran Jatinangor Indonesia; ^4^ Department of Anthropology Northwestern University Evanston Illinois USA; ^5^ Institute for Policy Research Northwestern University Evanston Illinois USA; ^6^ Queens College City University of New York New York New York USA; ^7^ New York Consortium in Evolutionary Primatology (NYCEP) New York New York USA; ^8^ Department of Anthropology Northern Arizona University Flagstaff Arizona USA; ^9^ Department of Anthropology University of Colorado Boulder Colorado USA; ^10^ Department of Anthropology University of Oregon Eugene Oregon USA

**Keywords:** Amazon, diastolic blood pressure (DBP), Ecuador, Epstein–Barr virus (EBV) antibodies, Shuar, somatic symptoms, stress, systolic blood pressure (SBP)

## Abstract

**Introduction:**

This study tests the hypothesis that self‐reported somatic symptoms are associated with biomarkers of stress, including elevated blood pressure and suppressed immune function, among Shuar adults living in the Ecuadorian Amazon.

**Methods:**

Research was conducted in three Shuar communities in the Upano Valley of the Ecuadorian Amazon and included the collection of biomarkers and a structured morbidity interview. Participants self‐reported somatic symptoms such as headaches, body pain, fatigue, and other bodily symptoms. We examined whether the number of somatic symptoms reported was associated with measures of immune (Epstein–Barr virus [EBV] antibodies) and cardiovascular (blood pressure) functioning in 97 Shuar adults (37 women, 60 men; ages 18–65 years). Multivariate linear regression analyses were used to examine the relationships among somatic symptoms and stress biomarkers, controlling for age, sex, body mass index (BMI), active infection, level of education, and income.

**Results:**

Controlling for relevant covariates, Shuar adults reporting the highest level of somatic symptoms (three symptoms) were more likely to exhibit elevated systolic (*β* = 0.20, *p* = 0.04) and diastolic blood pressure (*β* = 0.23, *p* = 0.03), in comparison to adults reporting no symptoms. Shuar adults reporting two symptoms, compared to no symptoms, were more likely to exhibit elevated EBV antibody concentrations (*β* = 0.34, *p* = < 0.01).

**Conclusions:**

These preliminary findings demonstrate that somatic symptoms reported by Shuar men and women are associated with physiological measures widely associated with chronic psychosocial stress. These findings complement the cross‐cultural literature in medical anthropology documenting the close connection between the expression of somatic symptoms and stressful life circumstances and highlight the important role that human biologists can play in exploring biocultural phenomena.

## 1 Introduction

“Somatic symptoms” is one of many terms (e.g., somatoform symptoms, psychosomatic symptoms, “medically unexplained” symptoms, and idioms of distress) that refers to a constellation of physical symptoms consistently associated with psychological stress (Kirmayer et al. 2004). Common somatic symptoms include headaches, insomnia, chronic pain, fatigue, blurred vision, and dizziness (Zijlema et al. 2013). While there are culturally‐specific expressions of somatic symptoms (Bagayogo, Interian, and Escobar 2013), a common feature across cultures is a connection between somatic symptoms and experiences of psychological distress (Koh 2018; Kirmayer, Dao, and Smith 1998). While there are plausible biological mechanisms underlying the experience of somatic symptoms that involve the physiological stress response (Baloh 2021a, 2021b), the question of whether individuals who express somatic symptoms also exhibit elevated markers of biological stress, remains largely unexplored.

Addressing this gap is important from a theoretical and practical perspective. On a theoretical level, much attention has been paid to the cultural, social, and psychological aspects of somatic symptoms by medical and cultural anthropologists. This line of inquiry began in earnest for anthropologists with Nichter's (1981, 402) research in South India where he observed that Bravhick women reporting somatic symptoms also reported significant sources of distress in their lives. Drawing from this analysis, Nichter (1981) coined the term “idioms of distress” to describe “an adaptive response or attempt to resolve a pathological situation in a culturally meaningful way”. Around the same time, medical anthropologist Arthur Kleinman found that many individuals presenting with neurasthenia in China, what would be considered to be depression in the United States, were responding to the cultural and physical harm inflicted upon them during the Chinese Cultural Revolution (Kleinman and Kleinman 1985). These studies spurred a larger research agenda, pushed forward primarily by medical anthropologists, which connected locally meaningful expressions of distress to larger political‐economic systems across multiple cultural contexts (Groleau and Kirmayer 2004; Kohrt and Hruschka 2010; Finerman 1989; Oths 1999; Guarnaccia and Farias 1988). While the majority of these studies proposed that somatic symptoms were bioculturally patterned illness experiences, there is a notable absence of any actual investigations of human biological variation in relation to the expression of somatic symptoms. This gap can be addressed by human biologists, and particularly by those employing a critical biocultural approach, which combines theory and methods from both the subdisciplines of human biology and critical medical anthropology to study the interface between biological and cultural factors affecting human health and well‐being (Baer 1996; Goodman and Leatherman 1998; Hruschka, Lende, and Worthman 2005; Leatherman and Goodman 2011).

Additionally, on a practical level, psychiatrists have historically been faced with challenges in differentiating somatic symptoms from depression or anxiety (Kapfhammer 2006) with continuing crossover between the diagnosis of somatization syndrome and other conditions such as hypochondriasis/illness anxiety, chronic pain, and medically unexplained syndromes (Rief and Martin 2014). While scholars across the social and medical sciences have generally gone beyond Cartesian notions of mind–body dualism, these splits often remain in Western scientific traditions and practices (Ecks 2009). Investigating whether somatic symptoms are associated with biological variation can potentially provide empirical evidence for the embodied nature of lived somatic experiences and, in the future, potentially be used to understand how somatic symptoms may be distinguished or related to other conditions.

In this paper, we test the hypothesis that self‐reported somatic symptoms are associated with biomarkers of stress among the Shuar of the Ecuadorian Amazonia. Shuar are a relatively large (> 100 000) Indigenous group centered primarily in the Morona‐Santiago region of Amazonian Ecuador (INEC 2023). As expanded upon by Liebert et al. (2025), the Shuar, and other Indigenous populations worldwide, are navigating substantial challenges related to colonization, land displacement, and poverty, which can produce substantial psychological distress (Madimenos et al. 2022; Snodgrass 2013; Valeggia and Snodgrass 2015). For example, Shuar have been exposed to rapid changes in market integration, defined as the suite of social and cultural changes that occur with economic development (Liebert et al. 2013). These processes have led to reduced participation in traditional subsistence activities, increased engagement with global economic markets, and more frequent interactions with non‐Indigenous mestizo neighbors (Blackwell et al. 2009). Human biologists working across cultures have shown that the myriad demographic, sociocultural, economic, and lifestyle changes associated with market integration have significant effects on chronic psychosocial stress, health, and biological functioning (Dressler 1999; Liebert et al. 2013; Mc Dade and Nyberg 2010; Snodgrass et al. 2007).

In this study, we build on this line of inquiry by investigating whether reports of somatic symptoms are correlated with elevations in Epstein–Barr virus (EBV) antibodies and blood pressure, among Shuar adults. Elevations in blood pressure (Dressler 1999) and in EBV antibody titers (McDade et al. 2000), are two well‐known and field‐friendly biomarkers of chronic psychological stress (DeCaro and Helfrecht 2022). EBV is a common herpes virus present in > 90% of adults worldwide (Macsween and Crawford 2003). Adequate cell‐mediated immune function generally maintains EBV in a latent state; however, immunosuppression linked to chronic stress causes the virus to reactivate, leading to a secondary humoral antibody response. Higher concentrations of EBV antibodies therefore indicate poorer cell‐mediated immune function associated with psychosocial stress (Glaser et al. 1991). Blood pressure is an indicator of both cardiovascular disease risk and psychological stress (Dressler and Bindon 2000). We hypothesize that higher levels of self‐reported somatic symptoms will be correlated with increased EBV antibodies and elevated blood pressure in Shuar adults.

## 2 Materials And Methods

### 2.1 Participants

This study was conducted in collaboration with the Shuar Health and Life History Project (www.shuarproject.org) and drew participants from three communities in the Upano Valley in the Ecuadorian Amazon. Individuals living in the Upano Valley have generally experienced higher levels of market integration than Shuar living in other regions (Liebert et al. 2013; Urlacher et al. 2016). Data were collected across a 2‐month period in a single field season in 2010. Biomarkers, anthropometrics, and morbidity data were available for 97 individuals between the ages of 18 and 65 years. All interviews were conducted in Spanish. Pregnant women were excluded from the study. All participants gave informed verbal consent, and the study protocol was approved by community leaders, the Northwestern University Institutional Review Board, the Office for Protection of Human Subjects at the University of Oregon, and the Federación Interprovincial de Centro Shuar.

### 2.2 Data Collection Procedures

A structured morbidity interview was administered to participants asking if they were experiencing any symptoms of sickness and, if so, to describe the symptoms. The morbidity interview was intended to elicit symptoms of active infection as the research project was primarily focused on documenting variation in C‐reactive protein (CRP) (a marker of acute inflammatory responses) and its association with symptoms of active infection (Mcdade et al. 2012). However, as the research team asked Shuar community members if they were sick, many of the reported symptoms fell outside of what is typically associated with an active infection (acute cough, fever, diarrhea etc.).

For example, a number of participants stated that they were experiencing dolor en mis organos or pain in their organs, mareos (dizziness), and tingling sensations in their hands and feet that pica (something that bites). They also reported escalofríos (chills), blackness and stars in their vision, and rapidly beating hearts. The printed structured morbidity interview only had three spaces to enter reported symptoms. The first author wrote down many additional symptoms along the sides of the survey, however, this approach was not consistently shared across the research team. When the first author returned from the field, she searched the literature to find an explanation for the clustering of noninfectious symptoms reported by Shuar community members. The literature that aligned best with these reports was the research on somatic symptoms and psychological distress.

As mentioned, the original structured morbidity survey had three spaces to enter reported symptoms. Thus, to maintain consistency for this analysis, only the symptoms written in these spaces were utilized to create a summary somatic symptom variable, biasing this study toward capturing the first three symptoms reported and underestimating the number of symptoms actually reported.

The symptom count variable constructed for this analysis included the symptoms utilized in the Somatic Symptom Severity Scale (PHQ‐15), which included stomach pain, chest pain, back pain, extremity pain (pain in arms and legs), joint pain, headaches, dizziness, heart palpitations, shortness of breath, fatigue/weakness, and gastrointestinal distress (constipation, loose bowels, or diarrhea—referred to as gastritis by Shuar). Thus, the PHQ‐15 was used as an initial codebook to categorize reported symptoms as somatic. However, the PHQ‐15 includes menstrual cramps and sexual intercourse pain, which were not reported in the sample and thus not a category included for our analysis. Furthermore, based on prior anthropological research on somatic symptoms, and the symptoms reported by the Shuar, we also included organ pain (such as pain in the liver, kidneys, and uterus), problems breathing, tingling sensations, and chills. In total, there were 17 symptoms that we categorized as somatic. However, the total somatic symptom count ranged from 0 to 3, reflecting the structure of the morbidity survey.

For indicators of chronic physiological stress, systolic and diastolic blood pressure were measured following standard guidelines (Chobanian et al. 2003). This involved using an automated blood pressure cuff (OMRON‐HEM 712C) to measure blood pressure while the individual was seated. Finger stick capillary whole blood samples were collected on filter paper (dried blood spots) and analyzed for EBV antibodies in the Laboratory for Human Biology Research at Northwestern University using an enzyme‐linked immunosorbent assay (DiaSorin #P001606A) and a protocol specifically validated for use with dried blood spot samples (McDade et al. 2000).

Relevant covariates were obtained through a structured interview and included demographic information such as age (years), sex (*male* = 1, *female* = 0), education level (years in school), and income (dollars per month). We also controlled for body mass index (BMI) and active infection. Two measures were used to assess the presence of active infection. First, data from the morbidity survey were used to create a symptom‐based measure of a cold‐like active infection. This was defined as reporting two or more symptoms that included cough, sore throat, runny nose, and mucus (*less than two symptoms* = 0, *two or more symptoms* = 1). Second, we measured elevated CRP from blood spots using an in‐house enzyme‐linked immunosorbent assay protocol specifically validated for use with dried blood spot samples (McDade, Burhop, and Dohnal 2004). CRP is an acute phase protein that is rapidly produced in response to active infection. Following previous work, a binary high CRP variable was defined as concentrations over 5.0 mg/L (McDade, Burhop, and Dohnal 2004). This cutoff value is based on the previous application of the dried blood spot CRP method and previous research in which plasma concentrations of CRP greater than 5–10 mg/L were associated with infection (McDade, Stallings and Worthman 2000). Prior analysis of matched plasma/blood spot samples showed that a blood spot CRP concentration of 5.0 mg/L is equivalent to 6 mg/L plasma CRP (McDade, Burhop, and Dohnal 2004). Our research with a subsample of participants from this study demonstrates no evidence of chronic low‐grade inflammation among the Shuar and elevated levels of CRP levels were associated with symptoms of active infection (McDade et al. 2012).

### 2.3 Statistical Analyses

All statistical analyses were conducted with Stata 17 (StataCorp, College Station, TX). Visual inspection of plotted histograms showed that EBV antibody concentrations and systolic and diastolic blood pressure were generally normally distributed. These biomarkers were analyzed as continuous dependent variables in separate multivariate linear regressions. Independent variables in each model included the number of somatic symptoms reported, age, sex, BMI, symptoms of active infection, elevated CRP, education, and income. These covariates were chosen because age, sex, and BMI are significant predictors of blood pressure (Dua et al. 2014), while under‐nutrition (determined by BMI) and active infection (elevated CRP and relevant symptoms) may influence immune function (EBV antibodies) (Chandra 1996). Income and education were included to control for the potential effects of socioeconomic status on stress and health (Tallman 2018). The continuous summary somatic symptom variable was broken down into three dummy variables (one symptom, two symptoms, three symptoms vs. zero symptoms) to examine the association with EBV antibodies and blood pressure per additional symptom. Results were considered statistically significant at *p* < 0.05.

## 3 Results

Descriptive statistics are presented in Table 1.

**TABLE 1:** Sample characteristics of Shuar adults (*n* = 97).

Measure	Mean (SD)	Median (range)
Age (years)	33.9 (11.2)	33.0 (18–65)
Monthly household income (dollars)	255.8 (305.4)	10.0 (0–600)
Schooling (years)	6.8 (3.9)	6.0 (0–17.0)
Height (centimeters)	153.1 (7.0)	152.5 (138.5–169.0)
Weight (kilograms)	60.0 (9.1)	59.3 (24.9–86.7)
Somatic symptoms^a^	1.18 (1.02)	1.0 (0–3)
Symptoms of active infection	0.6 (1.0)	0.0 (0–4.0)
C‐reactive protein (mg/L)	1.4 (4.4)	0.3 (0–31.8)
Epstein–Barr virus (ELISA units)	137.8 (66.9)	132.9 (27.4–281.2)
Systolic blood pressure (mm/Hg)	114. 6 (14.6)	113.0 (84.0–167.0)
Diastolic blood pressure (mm/Hg)	74.6 (9.2)	74 (49.0–104.0)

^a^ Summary: somatic symptom variable was constructed based on reports of the following symptoms: fatigue, headache, body pain, back pain, chest pain, extremity pain, joint pain, organ pain, stomach pain, nausea, gastritis, vision problems, dizziness, problems breathing, tingling, heart palpitations, and chills.

The median number of somatic symptoms reported in response to the structured morbidity interview was one symptom (range 0–3, Table 1). Table 2 details the percentages of the sample who reported zero, one, two, or three symptoms.

**TABLE 2:** Percentage of individuals who listed zero, one, two, or three somatic symptoms.

	All (*n* = 97)	Males (*n* = 37)	Females (*n* = 60)
Zero symptoms	33.0%	37.8%	30.0%
One symptom	25.8%	27.0%	25.0%
Two symptoms	28.9%	27.0%	30.0%
Three symptoms	12.4%	8.1%	15.0%

The most commonly reported somatic symptoms by participants were headache (23.5%), body pain (17.4%), stomach pain (12.2%), and organ pain (12.2%). 10% or less of the participant population reported fatigue, nausea, joint pain, and back pain. Less than 3% of participants reported vision problems, extremity pain, gastritis, chest pain, vision problems, dizziness, problems breathing, tingling, heart palpitations, and chills (Table 3).

**TABLE 3:** Percentage of participants reporting specific somatic symptoms.

	All (*n* = 97)^a^	Males (*n* = 37)	Females (*n* = 60)
Headache	23.5%	13.2%	30.0%
Body pain	17.4%	13.2%	20.0%
Stomach pain	12.2%	10.5%	13.3%
Organ pain	12.2%	7.9%	15.0%
Fatigue/weakness	10.2%	10.5%	10.0%
Nausea	10.2%	10.5%	10.0%
Joint pain	7.1%	7.9%	6.7%
Back pain	7.1%	7.9%	6.7%

^a^ 3% or less of the sample reported vision problems, extremity pain, gastritis, chest pain, vision problems, dizziness, problems breathing, tingling, heart palpitations, and chills.

Multivariate models controlled for covariates such as age, sex, BMI, CRP, and a symptom‐based measure of cold‐like active infection. As mentioned, the continuous summary somatic symptom variable was broken down into three dummy variables (one symptom, two symptoms, three symptoms vs. zero symptoms) to examine the association with EBV antibodies and blood pressure per additional symptom. Table 4 reports the results.

**TABLE 4:** Multivariate regression models examining the association between number of somatic symptoms reported (1, 2, and 3 symptoms compared to 0 symptoms) and systolic blood pressure, diastolic blood pressure, and Epstein–Barr Virus (EBV) antibodies (*n* = 97).

Systolic blood pressure				Diastolic blood pressure			EBV antibodies		
Variables	Coefficients (SE)	*β*	*p*	Coefficients (SE)	*β*	*p*	Coefficients (SE)	*β*	*p*
Somatic symptoms (1)	0.93 (3.29)	0.03	0.77	2.17 (2.29)	0.11	0.35	7.25 (17.02)	0.05	0.67
Somatic symptoms (2)	3.64 (3.32)	0.11	0.27	1.57 (2.31)	0.08	0.50	49.86 (17.15)	0.34	< 0.01*
Somatic symptoms (3)	9.31 (4.45)	0.20	0.04*	6.86 (3.10)	0.23	0.03*	26.63 (23.02)	0 0.13	0.25
BMI	1.40 (0 0.43)	0.30	< 0.01*	0.79 (0.29)	0.27	0.01*	1.13 (2.22)	0.05	0.61
Active infection	5.35 (3.55)	0.13	0.14	0.68 (2.47)	0.03	0.78	−11.90 (18.32)	0.07	0.51
CRP	−13.30 (6.50)	−0.18	0.04*	−3.26 (4.53)	−0.07	0.47	−11.46 (33.61)	−0.03	0.73
Age	0.22 (0.13)	0.16	0.09	0.12 (0.08)	0.14	0.14	1.40 (0.65)	0.24	0.04*
Male sex	−10.97 (2.58)	−0.36	< 0.01*	−4.19 (0.79)	−0.22	0.02*	8.95 (13.32)	0.07	0.50
Education	−0.30 (0.34)	−0.08	0.38	0.11 (0.24)	0.05	0.62	0 0.72 (1.77)	0.04	0.68
Income	0.01 (0.01)	0.09	0.28	0.01 (0.00)	0.14	0.17	−0.028 (0.06)	−0.05	0.63
Adjusted *R* ^2^	0.3207			0.1696			0.1101		

* *p* < 0.05.

To summarize, controlling for relevant covariates, Shuar adults reporting the highest level of somatic symptoms (three symptoms) were more likely to exhibit elevated systolic (*β* = 0.20, *p* = 0.04) and diastolic blood pressure (*β* = 0.23, *p* = 0.03), in comparison to adults reporting no symptoms. Shuar adults reporting two symptoms, compared to no symptoms, were more likely to exhibit elevated EBV antibody concentrations (*β* = 0.25, *p* = 0.02). Reporting three symptoms was not significantly associated with an increase in EBV antibody concentrations. Associations not related to the primary research questions included a positive association between systolic and diastolic blood pressure and BMI (*β* = 0.30, *p* = 0.001 and *β* = 0.27, *p* = 0.01, respectively) and being female (*β* = −0.36, *p* < 0.001 and *β* = −0.22, *p* = 0.02, respectively). Systolic blood pressure was inversely correlated with CRP concentrations (*β* = −0.18, *p* = 0.04).

Stratifying the regressions by sex shows a similar association between somatic symptoms and EBV for both men and women, with two somatic symptoms being significantly associated with higher EBV levels in each group. When examining stratified regressions for somatic symptoms and blood pressure, no significant results emerge, likely due to very small sizes (*n* = 37 women and *n* = 60 men). Finally, we ran the regressions with each individual symptom to examine whether there were certain symptoms driving the relationships between somatic symptoms and physiological functioning. While some symptoms were significant and others were not, a clear pattern of symptom clusters did not emerge.

## 4 Discussion

We found that Shuar adults reporting the highest level of somatic symptoms (three symptoms) were more likely to exhibit elevated systolic and diastolic blood pressure in comparison to adults reporting no symptoms. Shuar adults reporting two symptoms, compared to no symptoms, were more likely to exhibit elevated EBV antibody concentrations. However, reporting three symptoms was not significantly associated with an increase in EBV antibody concentrations. These results do not allow us to infer the causal direction of the associations between indicators of physiological stress and somatic symptoms. However, they do indicate that somatic symptoms are correlated with biological variation in immune and cardiovascular functioning among a population exposed to rapid lifestyle changes.

Our findings complement a large body of cross‐cultural work in the field of medical anthropology showing that somatic symptoms are linked to stressful social and political‐economic circumstances (Kirmayer, Dao, and Smith 1998). Specifically, medical anthropologists have explored how expressions of somatic symptoms may serve as idioms of distress (Nichter 1981), which are “culturally resonant means of experiencing and expressing distress in local worlds.” (Nichter 2010, 405). While the current study did not include in‐depth illness narratives to capture relevant idioms of distress, during our interview process study participants articulated connections between their physical suffering and difficulties making a living amidst significant environmental and social changes in the Ecuadorian Amazon. These observations align with Finerman's (1989) findings that Saraguro women in the Ecuadorian Andes related their experiences of nervios to shifts in gender roles and power that occurred with the migration of Saraguro men to the Amazonian lowlands to pursue agropastoralism. In their analysis, themes such as the relationship between power and powerlessness as well as gendered experiences of stress were connected to observations of how capitalistic market forces influence the flows of production and people. Oths (1999) and Carey (1993) also investigated culture‐bound illnesses in the Peruvian Andes and arrived at similar conclusions about the role of cultural change, psychological distress, and the expression of somatic symptoms.

This is the first study examining the expression of somatic symptoms in an Indigenous Amazonian population undergoing socioeconomic and cultural change.

Our findings that two somatic symptoms, versus three symptoms, were positively associated with EBV antibodies does not lend itself to a straightforward interpretation. This pattern of association may be the result of small sample sizes, the limitation of only examining up to three somatic symptoms (rather than the full breadth that were reported), or the inherent nature of the association. Additionally, this research was cross‐sectional, limiting our ability to comment on whether cultural change is at the root of the expression of somatic symptoms among Shuar. However, there is a large body of human biological research finding that EBV antibodies and blood pressure function as measures of chronic stress in populations exposed to lifestyle changes across the globe (McDade et al. 2000; McClure et al. 2010; Dressler 1991a, 1991b), with relevance for future work on this topic among Shuar.

For example, researchers working with the Samoan Migrant Project (Baker, Hanna, and Baker 1986) found that migrating to an urban environment, residing on a more “westernized” island, and living closer to an urban center, were associated with elevated blood pressure, poorer reported health, and increased concentrations of stress hormones, such as norepinephrine (Hanna and Fitzgerald 1993; Janes 1990; McGarvey and Baker 1979; Pearson, James, and Brown 1993). Since this early research, anthropologists have pushed forward more nuanced measures to understand the biological stress of shifting environments. Researchers have found that a lack of cultural consonance in lifestyle and lifestyle incongruity have been linked to elevated blood pressure, elevated EBV antibodies, depressive symptoms, and compromised psychological well‐being in individuals living in Samoa, St. Lucia, Siberia, Brazil, Bolivia, and the United States. (Bitton, McGarvey, and Viali 2006; Chin‐Hong and McGarvey 1996; Dressler 1991a, 1991b; Dressler et al. 2005; McDade 2001; Reyes‐García et al. 2010; Sorensen et al. 2009). Our study adds to this body of work with an explicit focus on the biological correlates of somatic symptoms among a population experiencing rapid lifestyle changes in the Ecuadorian Amazon and would be well accompanied by additional research collecting quantitative measures of psychological stress and qualitative data on the lived experiences of Shuar community members.

Outside of anthropology, there have been a few studies that have examined the relationship between the expression of somatic symptoms, blood pressure, and EBV antibodies, with generally inconclusive results. For example, Bayturan et al. (2022) did not find any relationship between EBV antibodies and the expression of somatic, or depressive, symptoms among children. Similarly, another study among adolescents, did not find any associations between EBV antibody levels and functional somatic symptom scores (Jonker et al. 2022). Mixed results have been found when examining the association between somatic symptom reporting and blood pressure. Kristal‐Boneh et al. (1998) found higher levels of somatic symptom reporting were associated with elevated systolic blood pressure, while Borres, Tanaka, and Thulesius (1998) reported that psychosomatic symptoms were associated with lower blood pressure among children. Despite the contradictory nature of these studies, scholars are still postulating that there may be shared biological mechanisms, primarily rooted in hypersensitization of the limbic system, which could potentially explain the experiences of those reporting somatic symptoms (Baloh 2021a). Our results, and this line of inquiry, may have implications for how psychiatrists characterize and treat what are often considered to be “medically unexplainable symptoms” (Baloh 2021b).

In addition to the main finding that reported somatic symptoms were associated with EBV antibodies, systolic and diastolic blood pressure, we found that EBV antibody concentrations were positively associated with age. This is in line with prior research documenting age‐related increases in EBV antibody titers (Schmader, van der Horst, and Klotman 1989). We also documented that females in this population had a higher BMI than males on average and that systolic and diastolic blood pressure were positively associated BMI. The positive association between BMI and blood pressure is well‐documented (Dua et al. 2014). While there is a large body of literature showing that men have higher blood pressure than women regardless of ethnicity (Sandberg and Ji 2012), recent research indicates that women exhibit a steeper increase in blood pressure throughout the life course compared to men (Ji et al. 2020). Finally, systolic blood pressure was inversely correlated with CRP concentrations in this study. This is contrary to most studies, which have found that CRP is associated with hypertension (Hage 2014).

This study has several limitations. First, a standardized somatic symptom inventory was not used, as the primary purpose of the original morbidity questionnaire was to elicit symptoms of active infection. As explained in the methodology, the structure of the morbidity questionnaire made it impossible to fully examine the substantial number of somatic symptoms reported by Shuar community members, in a manner conducive to a systematic analysis. Future research should utilize standardized methods such as the Patient Health Questionnaire (PHQ‐15), which includes symptom prompts, and should also provide open‐ended opportunities for study participants to speak about the range of symptoms they are experiencing (even when they go beyond validated surveys). Opening spaces for these discussions can allow researchers to more fully understand the number, severity, and context surrounding somatic symptoms expression. Second, no measures of reported psychological stress, nor ethnographic examinations of culturally‐specific idioms of distress, were obtained. The hypothesis that the symptoms reported by Shuar are associated with stress requires further research to determine if individuals reporting multiple somatic symptoms also experience a high degree of psychological stress. Finally, our sample size of 97 individuals is small, and the cross‐sectional design of the study limits discussions of the directionality of associations.

Future research should address these limitations and examine whether stressors differ in remote Shuar communities retaining traditional lifestyles in comparison to communities in the Upano Valley that are being exposed to rapid cultural and economic changes. More detailed studies of the histories, and lived experiences, of Shuar community members can open the doors to utilizing a critical biocultural approach to more comprehensively understand the meaning, and biological correlates, of somatic symptoms.

## 5 Conclusion

In conclusion, the results of this study contribute to research on stress and somatic symptoms in the fields of medical anthropology and human biology as well as to a small but growing body of literature suggesting that somatic symptoms are connected to biological functioning through a physiology of distress that may hold across varied socio‐ecological contexts (Dimsdale and Dantzer 2007). This perspective opens new doors for medical anthropologists and human biologists interested in using a critical biocultural approach to investigate the causes and consequences of somatic symptoms—as field‐friendly, minimally‐invasive approaches can provide a window into the biological correlates of stress for a wide variety of populations transitioning to market‐based economies.

## Author Contributions

**Paula S. Tallman:** conceptualization, data curation, investigation, writing. **Rebecca A. Seligman:** conceptualization, supervision, writing. **Felicia C. Madimenos** and **Tara J. Cepon‐Robins:** investigation, writing. **Melissa A. Liebert:** data curation, investigation, writing. **J. Josh Snodgrass, Thomas W. McDade** and **Lawrence S. Sugiyama:** funding, supervision, writing.

## Conflicts of Interest

The authors declare no conflicts of interest.

## Data Availability

Research data are not shared.
